# Italian Version of the Fundamentals of Care Framework and the Fundamentals of Care Practice Process: A Comprehensive Validation Study

**DOI:** 10.1111/jan.70099

**Published:** 2025-07-28

**Authors:** Annamaria Bagnasco, Maura Lusignani, Nicola Pagnucci, Talita Sallai, Gianluca Catania, Francesca Napolitano, Alberto Dal Molin, Beatrice Mazzoleni, Simone Cosmai, Daniela Cattani, Laura Mansi, Doriana Montani, Andreina Zavaglio, Paola Sanvito, Chiara Cartabia, Letteria Consolo, Luca Giuseppe, Milko Zanini, Loredana Sasso

**Affiliations:** ^1^ Department of Health Sciences University of Genoa Genoa Italy; ^2^ Department of Biomedical Sciences for Health, University of Milan Milan Italy; ^3^ Department of Translational Research and New Surgical and Medical Technologies University of Pisa Pisa Italy; ^4^ Policlinico San Martino Genoa Italy; ^5^ Department of Translational Medicine University of Piemonte Orientale, Maggiore Della Carità University Hospital Novara Italy; ^6^ Department of Biomedical Sciences Humanitas University Pieve Emanuele Italy; ^7^ IRCCS Humanitas Research Hospital Rozzano Italy; ^8^ Department of Translational Medicine University of Piemonte Orientale, Azienda Sanitaria del Verbano‐Cusio‐Ossola Vercelli Italy

**Keywords:** clinical practice, cultural adaptation, fundamentals of care, health systems, nursing education, person‐centred care, qualitative research, theoretical framework, translation

## Abstract

**Aims:**

To translate, culturally adapt and validate the Italian version of Fundamentals of Care Framework and the Fundamentals of Care Practice Process.

**Design:**

Qualitative tool validation study.

**Methods:**

The study followed internationally recommended procedures, including forward–backward translation, expert committee review, content validation through cognitive interviews and face validity testing with nurses and nursing students. Data were collected between January and October 2023.

**Results:**

Key terms were culturally and linguistically adapted to enhance clarity and contextual relevance, with changes informed by expert feedback. Content validation confirmed conceptual equivalence, and face validity testing demonstrated that Italian versions were perceived as clear, appropriate and applicable across clinical and educational settings.

**Conclusion:**

Cultural adaptation of theoretical frameworks is essential for ensuring their relevance and usability in local contexts. The Italian versions of the Fundamentals of Care Framework and the Fundamentals of Care Practice Process will provide a robust, evidence‐based foundation for person‐centred care across education, research and clinical practice.

**Impact:**

By making these tools accessible in Italian, this study supports the integration of fundamentals of care into national nursing education and practice, promoting international consistency in person‐centred care. It lays the groundwork for curriculum reform, clinical implementation and global collaboration in nursing.

**Reporting Method:**

Consolidated Criteria for Reporting Qualitative Research (COREQ) checklist.

**Patient or Public Contribution:**

This study did not involve any patient or public contribution.

**Trial Registration:**

ClinicalTrials.gov identifier: NCT05177627

**Summary**

- What is already known about the topic
  ○ The Fundamentals of Care Framework is an internationally recognised model for delivering person‐centred nursing care.
  ○ Cultural adaptation is essential to ensure the effectiveness of theoretical frameworks in different countries.
  ○ In Italy, validated versions of the Fundamentals of Care Framework and the Practice Process are not yet available.
- What this paper adds
  ○ Provides a culturally adapted and validated Italian version of both the Fundamentals of Care Framework and the Practice Process.
  ○ Demonstrates strong conceptual equivalence, clarity and applicability of the translated tools in Italian educational and clinical settings.
  ○ Offers a structured linguistic and cultural adaptation methodology that can be replicated for other frameworks or contexts.
- The implications of this paper for practice or policy
  ○ Supports the integration of the Fundamentals of Care into Italian nursing education and clinical practice.
  ○ Provides a foundation for improving person‐centred care and strengthening the delivery of fundamental nursing care nationally.
  ○ Facilitates international collaboration by aligning Italian nursing practice with globally recognised standards of care.

## 1 Introduction

Nursing is a profession grounded in both theory and practice, with nursing theories guiding values, beliefs, and approaches to care, and enhancing the quality of practice by helping nurses articulate interventions and their impact on patient outcomes (Younas and Quennell 2019). As the profession evolves, research has introduced various theories and models aimed at improving the quality of care. Among these, patient‐centred care has emerged in healthcare settings as a fundamental approach, emphasising the importance of addressing each patient's unique needs and potential (Edgman‐Levitan and Schoenbaum 2021). Within this paradigm, the Fundamentals of Care (FoC) Framework provides a structured model to integrate fundamental aspects of nursing care into practice, reinforcing the core principles of patient‐centred care (Kitson et al. 2013). Building on this framework, the Fundamentals of Care Practice Process (FoC‐PP) serves as a practical tool that translates the FoC Framework into clinical application (Rey et al. 2020).

However, while theoretical frameworks and practical tools offer essential guidance, their impact on clinical practice depends on their accessibility, applicability and ease of implementation (McArthur et al. 2021). Addressing this divide requires a multi‐faceted approach that considers the contextual healthcare environment and resource constraints (Freitas de Mello et al. 2024). To ensure that students and nurses can fully engage with the FoC Framework and the FoC‐PP, these need to be available in their local language (Muntlin and Jangland 2023). Without this, comprehension may be limited, hindering the framework's adoption, integration and effectiveness in daily practice. To support nursing leaders, educators and practitioners in effectively incorporating the FoC Framework into clinical settings, it is crucial to provide a linguistically and culturally adapted version. Doing so will not only enhance understanding and engagement but also strengthen the delivery of patient‐centred care, ultimately improving healthcare quality and outcomes.

## 2 Background

Ensuring patient safety and high‐quality care remains a significant challenge for nurses worldwide. Among the key barriers, inadequate nurse staffing has been consistently associated with adverse patient outcomes such as medication errors, falls, infections and increased mortality (Aiken et al. 2014; Griffiths et al. 2019; Lake et al. 2019; Blume et al. 2021). The findings of the Francis (2013) Inquiry in the UK further emphasised this issue, revealing that staffing shortages contributed to nurses' inability to provide fundamental care or challenge unsafe practices.

A related concern is the concept of *Missed Nursing Care—*the omission or delay of required care due to time constraints or limited resources—often involving essential activities like hygiene, mobilisation and emotional support (Kalisch 2006; Danielis et al. 2022; Gong et al. 2025). Findings from the RN4CAST study, including data from Italy, confirm that higher nurse workloads correlate with increased mortality and decreased patient satisfaction, reinforcing the importance of safe staffing and supportive practice environments (Aiken et al. 2014; Sasso et al. 2017).

Beyond structural deficits, a lack of role clarity and recognition of the essential nature of nursing care further complicates these challenges, highlighting the need for structured approaches to integrating fundamentals of care into everyday practice (Voldbjerg et al. 2017; Pene et al. 2025). Organisational ambiguity and limited managerial support reduce the integration of core nursing activities into daily practice and negatively impact on nurses' motivation, job satisfaction and retention (Mudd et al. 2023; Bagnasco et al. 2024). These issues underscore the urgent need for both structural and educational reforms, including the integration of the Fundamentals of Care framework into nursing education and clinical practice.

The Fundamentals of Care (FoC) Framework was developed in response to these challenges. It presents one of the most comprehensive conceptualisations of fundamental nursing care available, emphasising a holistic and integrated approach to care delivery rather than viewing it as a collection of isolated tasks (Feo, Conroy, et al. 2018; Feo, Kitson, and Conroy 2018). It outlines the dimensions needed to provide comprehensive, person‐centred care and offers a shared language for nurses to discuss and reflect on their practice, emphasising the complexity and importance of nursing care (Kitson et al. 2013). This framework highlights the nurse–patient relationship, the integration of physical, psychosocial and relational needs, and the context of care (Mudd et al. 2020). Developed by the International Learning Collaborative (ILC), a network of researchers, educators, clinicians and nursing leaders dedicated to improving fundamental care globally (International Learning Collaborative 2020) the framework has been implemented in several healthcare systems and educational settings (Jangland et al. 2016, 2018; Alderman et al. 2018; Voldbjerg et al. 2018, 2020; Muntlin et al. 2023; Waltrovitz et al. 2024).

As part of our initiative to embed the Fundamentals of Care (FoC) into the nursing curriculum, we identified both the FoC Framework and the FoC Practice Process (FoC‐PP) as essential tools to support students from their first year through clinical practice (Kitson 2020; Conroy et al. 2021). The FoC‐PP offers a structured model for delivering and improving care across settings and serves as a practical tool for both students and practitioners (Rey et al. 2020).

By incorporating a structured, evidence‐based approach to the fundamentals of care into the curriculum, we aim to support students in developing a deeper understanding of the FoC Framework and critically engage with its application (Feo et al. 2022). However, for their successful implementation, these tools must be accessible in the local language. Ensuring conceptual and linguistic equivalence is vital to effective implementation, cross‐cultural research and high‐quality care delivery (Sousa and Rojjanasrirat 2011).

While the FoC Framework has already been translated and culturally adapted in several languages including French, Danish, Norwegian, Portuguese, Spanish, Dutch, Swedish and Finnish, with all versions available on the ILC website, neither the FoC Framework nor the FoC‐PP have been translated into Italian. By translating and validating these resources in Italian, we aim to enhance their accessibility and relevance, facilitating their adoption across academic and clinical settings. This initiative bridges the gap between education and practice, reinforcing the centrality of fundamental nursing care in delivering high‐quality, person‐centred healthcare.

Despite the growing recognition in Italy of persistent challenges such as missed care and staffing strain, structured, theory‐informed approaches to fundamentals of care remain underused. Providing the Italian‐language versions of the FoC Framework and FoC‐PP addresses both linguistic and professional barriers—empowering educators, students and practitioners to engage more meaningfully with foundational nursing principles. Ultimately, this work supports national healthcare priorities by promoting evidence‐based, person‐centred care and advancing nursing workforce development.

## 3 The Study

### 3.1 Aim of the Study

To translate, culturally adapt and validate the Fundamental of Care Framework and the Fundamentals of Care Practice Process for the Italian context, making them accessible and applicable to the scientific and professional nursing community in Italy.

## 4 Methods

### 4.1 Design

This study employed an exploratory qualitative design to translate and culturally adapt the Fundamentals of Care (FoC) Framework and the Fundamentals of Care Practice Process (FoC‐PP) into Italian. The process followed the guidelines recommended by Beaton et al. (2000), Sousa and Rojjanasrirat (2011), and an overview of the adaptation steps is provided in Table 1. These approaches offer structured procedures to ensure linguistic equivalence, conceptual clarity and cultural appropriateness across translated instruments, and which have been successfully applied in previous adaptations of the FoC Framework, including the Swedish version (Muntlin et al. 2023).

**TABLE 1:** Overview of the translation and cultural adaptation process adapted from Beaton et al. (2000) and Sousa and Rojjanasrirat (2011).

Phase	Description	Methodological basis	Key steps
1. Translation & Cultural Adaptation	Forward‐backward translation of FoC Framework and FoC‐PP into Italian	Beaton et al. (2000); Sousa and Rojjanasrirat (2011)	– Independent forward translation (2 native Italian speakers) – Reconciliation into a single version – Back‐translation (native English speaker) – Review by expert committee for cultural/contextual appropriateness
2. Content Validity Testing	Expert evaluation using cognitive interviewing	Sousa and Rojjanasrirat (2011); Güss (2018)	– Think‐Aloud technique – Semi‐structured interviews – Focus on item clarity, terminology, conceptual alignment
3. Face Validity Testing	User testing with students and practicing nurses	Beaton et al. (2000); Sousa and Rojjanasrirat (2011)	– Survey via Microsoft Forms – Target: comprehension, word complexity and cultural appropriateness – 20 participants (students and nurses)

### 4.2 Sample and Data Collection

The original English version of the FoC Framework version was obtained from the ILC webpage with content drawn from Kitson et al. (2013) and Feo et al. (2017). The original English version of the FoC‐PP was obtained from Feo et al. (2017). After obtaining formal permission from the ILC, the translation and validation process was conducted between January and October 2023, encompassing three sequential phases: translation and cultural adaptation (Phase 1), content validity testing (Phase 2) and face validity testing (Phase 3).

### 4.3 Phase 1: Translation and Cultural Adaptation

The translation and cultural adaptation process adhered to established guidelines from both Beaton et al. (2000) and Sousa and Rojjanasrirat (2011) to ensure linguistic accuracy, conceptual equivalence and cultural relevance. The translation involved a forward‐backward translation process. Two native Italian speakers independently translated the FoC Framework and the FoC‐PP from English to Italian. These translations were merged to create the first reconciled Italian versions, ensuring a comprehensive synthesis of both interpretations. To assess conceptual equivalence, a native English speaker back translated the reconciled Italian version into English. This back‐translation served as a validity check, allowing for the identification of any discrepancies between the source and the translated versions.

To ensure the cultural and contextual appropriateness of the translated materials, a cultural adaptation committee composed of four members was established. The committee consisted of four members—independent from the translation team—to maintain methodological rigour. All members were involved in a broader initiative to integrate the FoC Framework and FoC‐PP into Italian nursing education and possessed substantial expertise in the field of fundamentals of care. The group included a nursing professor with extensive experience in nursing research and curriculum development, a senior lecturer specialising in nursing education and practice, an MSN student who also serves as a lecturer with clinical and academic expertise, and a PhD student with expertise in instrument validation. The committee reviewed the back‐translated and reconciled Italian versions to assess clarity, cultural relevance and alignment with Italian nursing terminology and practice. Key terms were selected for clarity and comprehension across educational levels and care settings. Where appropriate, certain English terms commonly used in Italian nursing discourse were retained to preserve familiarity and professional coherence.

### 4.4 Phase 2: Content Validity Test

Content validity was assessed through cognitive interviewing with a panel of experts in nursing education and research, using the ‘Think‐Aloud’ technique (Güss 2018). This method allowed participants to verbalise their real‐time cognitive processes while reviewing the translated instruments, offering insight into their interpretation of wording, structure and conceptual alignment. It aligns with cross‐cultural adaptation guidelines by Beaton et al. (2000) and Sousa and Rojjanasrirat (2011), which emphasise the importance of expert evaluation in establishing conceptual equivalence and linguistic clarity.

A qualitative approach was selected over a Content Validity Index (CVI) to allow a more nuanced exploration of participant reasoning and contextual considerations in adapting the instruments for the Italian setting.

A convenience sample of five researchers and educators with expertise in fundamental nursing care was recruited via email invitation. All participants provided a written informed consent, with verbal confirmation obtained at the start of each session to enable audio and video recording. Interviews were conducted via Microsoft Teams at times chosen by the participants. Each session lasted approximately 30 min.

Two trained female interviewers—one MSN student and lecturer with experience in qualitative methods, and one PhD student with expertise in instrument validation—facilitated the sessions. None of them had a prior relationship with participants.

Participants were introduced to the Think‐Aloud method and asked to verbalise their thinking processes while reviewing each item of the translated FoC Framework and FoC Practice Process. Interviewers intervened only when necessary—for example, during prolonged silences. Following this, a short semi‐structured interview was conducted to capture additional reflections on the instruments' completeness, usability and cultural appropriateness. The interview guide used in this phase is presented in Table 2.

**TABLE 2:** Cognitive interview guide for content validity.

Interview guide
Can you share your thoughts while reading each word of the FoC Practical Process? How would you modify the translation of the FoC Practical Process to make it more accessible to nurses and students? Can you share your thoughts while reading each word of the FoC Framework, particularly regarding its dimensions (relationship, integration of care and context of care)? What thoughts come to mind as you move from one dimension to another? How would you modify the translation of the FoC framework to make it more accessible to nurses and students? Do the Italian versions encompass all the relevant constructs and elements of the originals? Does the translated framework, in its entirety, reflect fundamental nursing care? In your opinion, how could the Italian translation of the framework and the practice process be effectively used by Italian students and nurses? How appropriate are those instruments for nurses within the Italian context?

### 4.5 Phase 3: Face Validity Test

To assess the clarity and accessibility of the Italian versions of the instruments, face validity testing was conducted with a group of 20 participants, including registered nurses and nursing students. This approach ensured feedback from a diverse range of end users with varying levels of experience.

A convenience sampling method was employed, with participants recruited in person during pre‐ and postgraduate lectures at an Italian university. Eligible participants were students enrolled in bachelor's and master's degree nursing courses. Participation was voluntary, and informed consent was obtained before data collection.

Data were gathered through an anonymous online survey administered via Microsoft Forms. The survey focused on evaluating word complexity, text comprehension and the identification of any inappropriate or potentially offensive terms. This process followed the guidelines of both Sousa and Rojjanasrirat (2011) and Beaton et al. (2000), which advocate pre‐testing with representatives of the target population to evaluate linguistic clarity, usability, and relevance of translated instruments.

### 4.6 Data Analysis

#### 4.6.1 For Phase 2

To ensure a rigorous evaluation, cognitive interview data were first transcribed and systematically analysed using a structured matrix approach described by Knafl et al. (2007). Think‐Aloud data were summarised for each item along with an inductively derived categorisation of the types of problem noted, focusing on expert feedback related to clarity, conceptual equivalence and relevance. Each item was entered as a separate row in the matrix, allowing for detailed, item‐by‐item comparison, refinement and tracking of suggested changes.

Semi‐structured interviews were conducted to evaluate the conceptual completeness and content validity of the translated tools. Selected participant quotations are presented in the Results section to illustrate expert consensus on the conceptual completeness and applicability of the translated tools. Based on these findings, the cultural adaptation committee incorporated expert recommendations, which led to the final Italian version of the FoC Framework and FoC‐PP. While the primary analysis focused on item‐level validation, broader reflections on the instruments' usability and cultural relevance were also gathered during these interviews.

#### 4.6.2 For Phase 3

Survey data from the face validity phase were analysed descriptively using frequencies and percentages. The analysis focused on participants' assessments of word complexity, ease of comprehension, and the identification of any potentially unclear or culturally inappropriate terms in the translated instruments.

### 4.7 Ethical Considerations

This study was approved by the Territorial Ethics Committee of the Liguria Region September 28, 2021 (501/2021—DB id. 11882) as part of the study ‘Integrating the Fundamentals of Care Framework into Italian Nursing Education (FOC‐FORM Study)’. All participants were fully informed about the study's purpose, procedures, potential risks and benefits before their involvement. Written informed consent was obtained from each participant to confirm their agreement to participate. For interviews requiring audio or video recording, verbal consent was additionally obtained at the beginning of the session.

Participation in the study was entirely voluntary, with participants informed of their right to withdraw at any time without consequences. Confidentiality and anonymity were ensured by assigning codes to interview transcripts and anonymising questionnaire responses. No money or incentives were provided for participation.

Data were securely stored and used exclusively for research purposes, in compliance with data protection regulations. Participants could ask questions and seek clarification before data collection began. The study adhered to the principles outlined in the Declaration of Helsinki and relevant national ethical guidelines, ensuring the highest standards of research integrity. Permission to use and reproduce material from external sources was obtained, with relevant citations provided in the manuscript.

### 4.8 Rigour

To ensure methodological rigour and trustworthiness, we followed the guidance outlined by Luciani et al. (2019) and the principles of qualitative research quality appraisal. Specifically, we addressed the four key dimensions of trustworthiness—credibility, dependability, confirmability and transferability—through a combination of purposeful design, reflexive awareness and transparent validation procedures.

Credibility was ensured through triangulation across multiple expert roles, including nursing educators, clinical nurses and students from diverse settings. This diversity enabled us to capture a range of perspectives and assess the relevance and clarity of the translated tools across user levels. The iterative nature of the content validation and face validity processes further contributed to internal consistency and confidence in the findings.

Dependability was supported by strict adherence to established cross‐cultural adaptation methodologies (Beaton et al. 2000; Sousa and Rojjanasrirat 2011). We followed a consistent protocol involving translation, reconciliation, expert review and matrix‐based analysis, which allowed for replicability and methodological stability.

Confirmability was addressed by maintaining detailed documentation throughout the translation and adaptation processes. This included decision logs, justification for terminology choices and the use of a structured matrix to systematically track expert feedback and resulting revisions. The cultural adaptation committee functioned as an independent oversight body, promoting reflexivity and guarding against individual bias.

Transferability was considered through the purposeful inclusion of participants from various roles and educational backgrounds, in line with sampling strategies recommended by Luciani et al. (2019). By engaging professionals from different practice areas, we enhanced the contextual relevance and potential applicability of the adapted tools across the Italian healthcare and educational system.

Finally, the study followed the Consolidated Criteria for Reporting Qualitative Research (COREQ) checklist (Tong et al. 2007; Table S1) to ensure transparency and rigour in the reporting of qualitative methods.

## 5 Results

### 5.1 Phase 1: Translation and Cultural Adaptation

The two independent forward translations were largely consistent but revealed differences in several key terms, including ‘Focus’, ‘Anticipate’, ‘Know’, ‘Evaluate’, ‘Quality and Safety’, ‘Governance’, and ‘Regulation and Accreditation’. These differences were reconciled into a merged version, which was then back translated into English to ensure conceptual alignment with the original text.

The cultural adaptation committee reviewed the reconciled version and made final adjustments to ensure clarity, contextual relevance and linguistic appropriateness within the Italian nursing context. Some English terms were retained due to their common usage in Italian professional practice. The final decisions on the terminology are presented in Tables 3 and 4.

**TABLE 3:** Key terminology adapted during Phase 1 of the FoC Framework translation.

Original term	Reconciled Italian version	Backtranslation	Final first version (Italian)	Final first version (English equivalent)
Focus	Centralità	Centrality	Focalizzarsi	Focusing
Anticipate	Anticipazione	Anticipation	Anticipare	Anticipate
Know	Conoscenza	Knowledge	Conoscere	Know
Evaluate	Valutazione	Evaluation	Valutare	Evaluate
Privacy	→	→	→	→
Comfort	→	→	→	→
Having values and beliefs considered and respected	Avere i valori e le credenze considerate e rispettate	Having values and beliefs considered and respected	→	→
Emotional wellbeing	Benessere emozionale	Emotional wellbeing	→	→
Helping patients to cope	Aiutare i pazienti ad adattarsi	Helping patients adapt	→	→
Engaging with care recipients	Impegnarsi con i pazienti	Engaging with care recipients	→	→
Supporting and involving families and carers	Sostenere e coinvolgere la famiglia e i caregivers	Supporting and involving families and carers	→	→
Working with patients to set goals	Stabilire gli obbiettivi assieme ai pazienti	Setting goals togheter with patients	→	→
Active listening	Ascoltare attivamente	Listening actively	→	→
Helping patients to stay calm	Aiutare i pazienti a mantenere la calma	Helping patients keep calm	→	→
Policy level	Livello Politico	Policy level	→	→
System Level	Livello di Sistema	System Level	→	→
Leadership	→	→	→	→
Evaluation and feedback	Valutazione e feedback	Evaluation and feedback	→	→
Quality and safety	Programmi per la qualità e la sicurezza	Quality and safety priorities	Qualità e sicurezza	Quality and safety
Governance	Processo di management e responsabilità	Management processes and accountability	Processi di governo e responsabilità	Governance Processes and Accountability
Financial	Risorse economiche	Financial resourses	Risorse economiche	Economic Resources
Regulation and accreditation	Regole e accreditamento	Rules and accreditation	Regolamentazione e accreditamento	Regulation and accreditation
Care recipient needs	Bisogni di chi riceve le cure	Needs of the care recipient	→	→
Personal cleansing and dressing	Igiene personale e vestizione	Personal hygiene and clothing	→	→
Medication management	Gestione della terapia	Drug management	→	→
Caregiver actions	Azione di chi eroga le cure	Action of healthcare provider	→	→

*Note:* → indicates that the term was maintained without modification across versions.

**TABLE 4:** Translation and cultural adaptation words of the FoC Practice Process.

Original term	Reconciled Italian version	Backtranslation	Final first version (Italian)	Final first version (English equivalent)
Fundamentals of Care practice process: 5 steps	Processo pratico delle Cure Fondamentali: 5 fasi	Fundamentals of Care Practice Process: 5 phases	→	→
1. Concepts: observation and reflection Ideas and opinions Facts Tacit knowledge	1. Concetti: osservazione e riflessione Idee e opinioni Fatti Conoscenza tacita	1. Concepts: observation and reflection Ideas and opinions Facts Tacit knowledge	→	→
2. Working hypothesis	2. Ipotesi di lavoro	2. Working hypothesis	→	→
3. The Fundamentals of Care Framework	3. Framework delle Cure Fondamentali	3. The Fundamentals of Care Framework	→	→
4. Theories	4. Teorie	4. Theories	→	→
5. Clinical‐reasoning Process	5. Processo di Ragionamento‐Clinico	5. Clinical‐reasoning Process	→	→

*Note:* → indicates that the term was maintained without modification across versions.

### 5.2 Phase 2: Content Validity Test

Five experts participated in the content validity assessment. The majority were females (80%, *n* = 4), with a mean age of 49.2 years (SD = 6.24). All participants were lecturers in undergraduate and postgraduate nursing education with extensive clinical backgrounds. Their academic qualifications included two Masters of Science in Nursing (MSN), one PhD in progress and two completed PhDs.

The analysis was conducted in two phases. First, using a structured matrix approach, expert feedback was reviewed item‐by‐item to assess clarity, conceptual equivalence and cultural relevance. We identified issues regarding ambiguous terminology, inconsistent tone and expressions poorly aligned with Italian nursing practice. These informed the final revisions made to the Italian versions of the instruments (summarised in Table 5).

**TABLE 5:** Refinement of terms based on expert feedback (content validity testing).

Initial term (from Phase 1)	Initial English equivalent	Expert feedback	Issue identified	Final adapted term (Italian)	Final English equivalent
Focalizzarsi	Focusing	‘It's rarely used in Italian… “Concentrarsi” feels more natural.’ (Expert 3)	Limited use of ‘focalizzarsi’ in Italian language	Concentrarsi	Focusing
Avere i valori e le credenze considerate e rispettate	Having values and beliefs considered and respected	‘The verb “to have” doesn't sound right… values and beliefs should be considered and respected.’ (Expert 1)	Repetition of the concept of ‘Respect’. ‘Consideration’ proposed as a synonym.	Considerazione dei valori e delle credenze	Consideration of values and beliefs
Benessere emozionale	Emotional wellbeing	‘“Emozionale” sounds like stress… prefer “benessere emotivo”’. (Expert 1)	Experts used ‘emotivo’ and ‘emozionale’ as synonyms, but favoured ‘emotivo’.	Benessere emotivo	Emotional wellbeing
Aiutare i pazienti ad adattarsi	Helping patients adapt	‘“Adattarsi” feels passive… “coping” implies active engagement’. (Expert 5)	Difficulty translating ‘to cope’—‘Adattarsi’ viewed as too passive	Essere di supporto	To be supportive
Impegnarsi con i pazienti	Engaging with care recipients	‘“Impegnarsi” implies personal effort, not shared engagement… “Coinvolgersi” is more accurate’. (Expert 4)	‘Impegnarsi’ not clearly understood; interpreted as individual commitment, not relational involvement	Coinvolgere i pazienti	Engaging with care recipients
Ascoltare attivamente	Listening actively	‘Yes, it could be “ascolto attivo”’. (Expert 1)	‘Ascolto attivo’ is more natural and standard terminology in Italian health communication literature.	Ascolto attivo	Active listening
Aiutare i pazienti a mantenere la calma	Helping patients keep calm	‘“Calm” sounds momentary… aim is to help reflection and self‐regulation’. (Expert 1)	‘Mantenere la calma’ seen as short‐term, not relational or sustained	Aiutare I pazienti a sentirsi tranquilli	Helping patients feel calm
Processi di governo e responsabilità	Governance Processes and Accountability	‘Governance is hard to translate—it includes leadership and accountability’. (Expert 4)	No exact Italian equivalent; ‘governance’ preferred due to conceptual breadth and familiarity in healthcare and academic contexts.	Governance	Governance
Livello politico	Policy level	‘It's difficult to truly translate the word “policy” into Italian, because “politico” doesn't really hold up as a term. The Italian translation of “policy” doesn't fully capture its meaning, which is much broader, better to keep “politiche”’ (Expert 4)	‘Livello politico’ interpreted as political rather than policy‐related	Politiche	Policies
Livello di Sistema	System Level	‘“Livello” is unclear… I prefer a broader context like “sistema” or “caratteristiche del sistema”’. (Expert 2)	‘Livello’ not meaningful to experts; preference for broader system context	Sistema	System

For example, the term ‘focalizzarsi’ was considered uncommon in Italian clinical contexts and was replaced with ‘concentrarsi’, which conveys the meaning of ‘focusing’ but aligns better with commonly used professional language and the intended action in nursing practice. Likewise, although both ‘impegnarsi con i pazienti’ and ‘coinvolgere i pazienti’ can be translated as *engaging with care recipients*, the former was considered to convey a more individualistic focus. In contrast, the latter more accurately reflects the mutual, relational emphasis that is fundamental to the framework.

The phrase ‘aiutare i pazienti ad adattarsi’, meaning *helping patients adapt*, was also reconsidered, as the term ‘adattarsi’ was perceived as overly passive and misaligned with the intended concept of coping. Experts suggested a more active and empowering alternative: ‘essere di supporto’, which can be translated as *to be supportive*, meaning *helping patients to cope* rather than simply adapt.

Structural terms such as *policy level* and *system level* were challenging to translate, as the word *level* did not align with natural Italian usage and was perceived as redundant. As a result, we decided to omit *level* and adopted the terms *‘politiche’* and *‘sistema’*, which ensure both conceptual clarity and linguistic appropriateness.

Notably, several terms flagged during the expert validation phase had already been identified as critical during the initial translation, reconciliation and back‐translation process. Their reappearance during expert validation confirmed the value of earlier adjustments and underscored the consistency and robustness of the adaptation strategy.

These and other key revisions are summarised in Table 5, showing the original terms, expert opinions, the nature of each issue and the final validated Italian terms.

In addition to the item‐level analysis, experts were invited to share their overall impressions of the instruments, including perceptions of their usability, conceptual completeness and cultural fit. The final phase of the analysis sought to confirm the content domain coverage of the translated instruments. Following Grant and Davis (1997), a holistic assessment was used to establish whether the tools adequately reflected the construct of fundamentals of care, beyond item agreement alone. Experts agreed that Italian versions preserved the integrity and intent of the original tools, ensuring their appropriateness for use in Italian nursing education and practice. Table 6 presents key insights from expert interviews that expand upon the comprehensive assessment of the Italian FoC Framework and FoC‐PP. These reflections address not only the conceptual clarity and cultural relevance of the translated tools, but also their perceived value in supporting fundamental nursing care across clinical practice, education and the formation of professional identity.

**TABLE 6:** Key insights from expert interviews on the Italian FoC Framework and FoC‐PP.

Conceptual focus	Illustrative quotes	Key insights
Clarity and fidelity of translation	‘You stuck to the literary text… it's very clear’. (Expert 4) ‘I found [all constructs] in the Italian version’. (Expert 5)	The Italian versions retained all key constructs and were perceived as conceptually faithful and clear.
Alignment with core nursing values	‘It brings forward a concept at the heart of nursing care’. (Expert 3) ‘They are the core needs internalised in our education’. (Expert 4)	The framework strongly resonates with nursing's fundamental values and holistic focus.
Nursing‐Care Model Relationship	‘If a nurse asks, “What is our unique contribution?”, they should refer to the FoC’. (Expert 3) ‘It focuses on who will be beside the patient’. (Expert 4)	The framework supports reflective, values‐based nursing practices and reinforces the relational role of nurses.
Clinical feasibility and realism	‘Nurses have become more technical… less empathetic’. (Expert 4) ‘Nurses should gather needs and delegate as necessary’. (Expert 4)	The FoC‐PP is seen as a feasible, structured tool that accommodates current clinical constraints while promoting shared responsibility and empathy.
Educational implementation	‘First‐year students need to understand why these elements matter’. (Expert 3) ‘Discrepancies arise if clinical instructors don't follow the framework’. (Expert 4)	Effective educational integration depends on alignment between theory and clinical practice.
Professional identity	‘It emphasises the importance of relationships in nursing’. (Expert 3) ‘We've lost touch with the essence of our profession’. (Expert 4)	Both tools help reaffirm professional identity by balancing technical and relational competencies.

### 5.3 Phase 3: Face Validity Test

The sample predominantly consisted of females, with a mean age of 33.05 years (standard deviation: 9.63 years). Half of the participants were clinical practice nurses, while a quarter were students in the Bachelor of Science in Nursing programme, two were nursing educators, two were nursing managers and one was a nursing researcher. The average length of service was 11.08 years (standard deviation: 10.54 years).

In terms of educational qualifications, five participants reported having a high school diploma, six were nurses with a bachelor's degree, eight had nursing specialty degrees and one held a master's degree in nursing and midwifery sciences.

**FIGURE 1:**
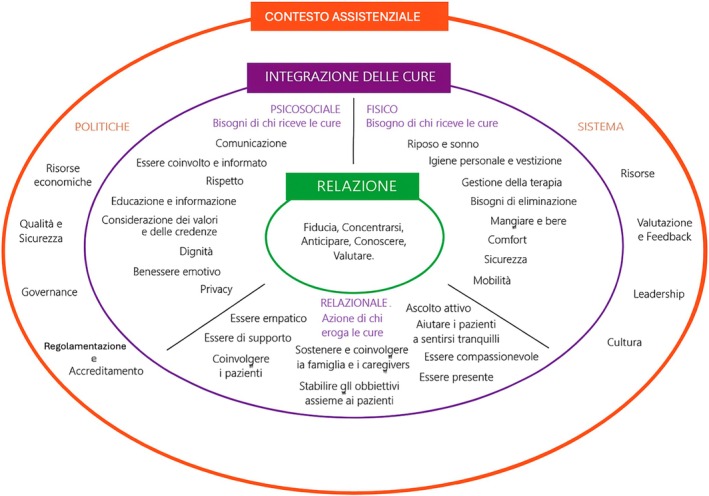
The Italian version of the Fundamentals of Care Framework.

**FIGURE 2:**
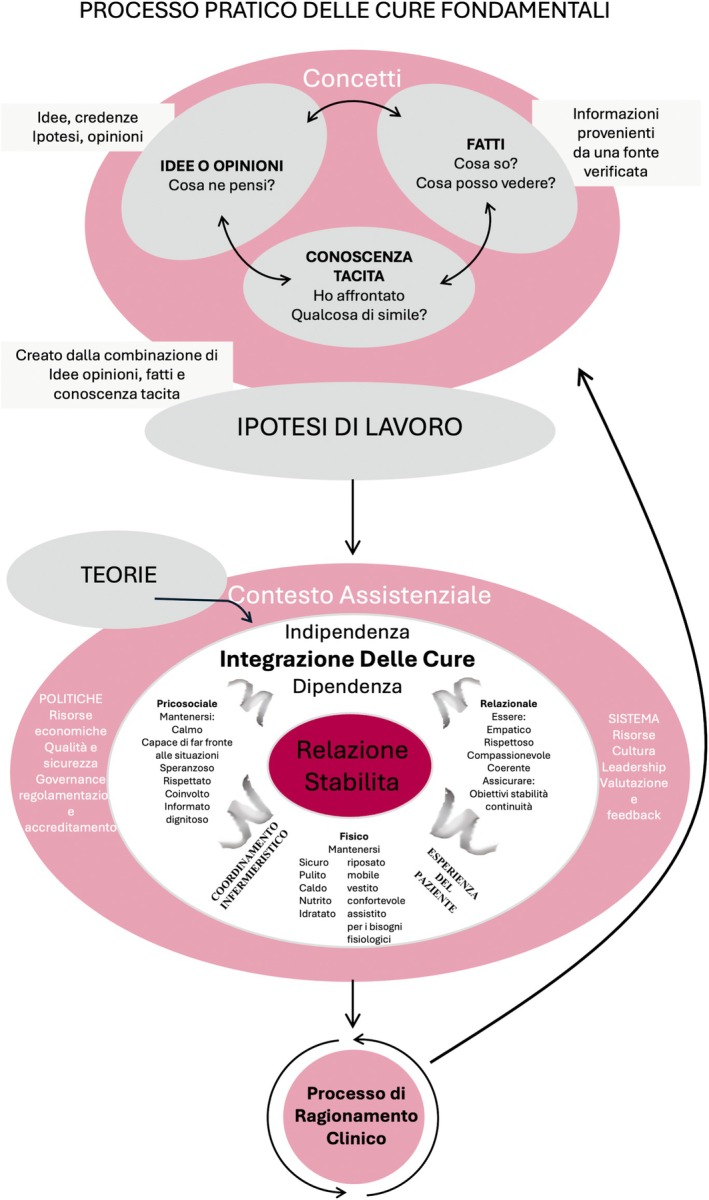
The Italian version of Fundamentals of Care Practice Process.

Regarding face validity, 95% of the participants found the language used in the framework easy to understand. All participants (100%) indicated that the wording was neither confusing nor offensive. Only one participant suggested rephrasing the term *Accreditation*, recommending it be revised to better reflect the concept of *conferring authority*. However, the term was retained, as this item had already obtained excellent content validity during Phase 2 of the validation process. Demographic characteristics of the participants involved in the face validity assessment are presented in Table 7. The final Italian versions of the FoC Framework and the FoC Practice Process, following translation, cultural adaptation and validation, are shown in Figures 1 and 2.

**TABLE 7:** Demographic characteristics of face validity participants (*N* = 20).

Variable	Category	*n*	%/M (SD)
Gender	Female	15	75%
	Male	5	25%
Age	Mean (SD)		33.05 (9.63)
Professional role	Clinical nurse	10	50%
	Nursing student (Bachelor's)	5	25%
	Nurse educator	2	10%
	Nurse manager/organisational role	2	10%
	Nurse researcher	1	5%
Years of experience	Mean (SD)		11.8 (10.54)
Educational qualification	High school diploma	5	25%
	Bachelor's degree (BSc Nursing)	6	30%
	Postgraduate Diploma	8	40%
	Master's degree (MSc Nursing/Midwifery)	1	5%
	PhD	0	0%

## 6 Discussion

This study presents the first dual validation of both the FoC Framework and Practice Process in the Italian context, advancing the international adaptation of person‐centred care instruments and addressing key gaps in implementation research. The translation and adaptation process adhered to internationally recognised guidelines, ensuring a high degree of fidelity to the original instruments while accommodating the specific linguistic and cultural nuances of the Italian context. The forward‐backward translation method, combined with expert consultation, proved effective in resolving discrepancies in terminology, such as *focus, governance, regulation* and *accreditation*. These terms, crucial for preserving conceptual clarity, were carefully refined to align with Italian nursing terminology and practices.

Terminological refinements were not merely linguistic but conceptual, reflecting the need to maintain integrity while ensuring relevance and usability in Italian professional and educational contexts. The recurring discussion around terms such as *engaging, to cope, system* and *policy level* revealed deeper interpretative tensions—where literal translations risked diluting core concepts like engagement, empowerment or structural responsibility. A similar nuance emerged in the revision of *helping patients keep calm*, which was perceived as a short‐term, almost disciplinary action. The revised phrasing, *helping patients feel calm*, enabled better capture of the intended emotional support and aligned more closely with the relational ethos of the Framework. This seemingly small change reflects a deeper cultural understanding of the nurse–patient dynamics and illustrates how linguistic nuances shape relational care. It also underscores the interpretative work required to balance fidelity to the original language with the conceptual clarity needed for local relevance.

The findings confirm the FoC Framework's theoretical robustness and cross‐cultural applicability, consistent with Sousa and Rojjanasrirat's (2011) assertion that maintaining conceptual equivalence is essential for the international adaptation of research instruments. The strong alignment observed between the original and translated versions confirms the FoC framework's global relevance for guiding nursing care.

In the Italian healthcare setting—characterised by workload pressures, role ambiguity and fragmented care processes—the FoC framework's holistic emphasis is particularly timely. Experts in this study confirmed the framework's conceptual completeness and its alignment with core nursing values, including person‐centredness, relational engagement and clinical reasoning (Feo, Conroy, et al. 2018; Feo, Kitson, and Conroy 2018; Kitson et al. 2013). Moreover, the framework's integration of physical, psychosocial and relational dimensions resonates with recent shifts in Italian nursing education, where holistic care models are being emphasised to address gaps between theory and practice and strengthen professional identity (Bagnasco et al. 2024; Feo et al. 2022).

In parallel, the FoC Practice Process extends the utility of the framework by operationalising its principles into everyday care. While the framework provides a conceptual foundation, the FoC‐PP translates it into a practical, step‐by‐step guide that supports nurses in recognising patient needs, building therapeutic relationships and delivering integrated care (Feo et al. 2017; Rey et al. 2020).

Expert feedback highlighted the FoC‐PP's relevance in addressing persistent challenges within Italian clinical settings, such as time constraints and increasing specialisation. Participants noted that relational aspects of care are often deprioritised as nurses focus on technical and administrative tasks. The FoC‐PP was seen as a means to contrast this trend by supporting prioritisation, delegation and shared responsibility. These features were considered critical for sustaining person‐centred care, particularly when one nurse cannot meet all the patients' needs alone. By fostering clinical reasoning and team coordination, the FoC‐PP may enhance the feasibility of delivering holistic care within complex health systems. Nonetheless, concerns remain about its routine implementation. Future research should explore how the FoC‐PP can be integrated into daily workflows without increasing documentation burdens or contributing to perceptions of added complexity.

Face validity testing further confirmed the accessibility and clarity of the Italian versions among students and practising nurses. Participants consistently reported that the language was understandable and culturally appropriate. While one participant suggested rephrasing the term *Accreditation*, the committee retained the original translation due to its recognised role in nursing governance and its prior validation during Phase 2. This decision illustrates the balance between cultural adaptation and conceptual fidelity.

Beyond confirming conceptual fidelity and cultural relevance, this work offers a practical blueprint for integrating person‐centred care principles into national education and practice standards. Situated within a broader policy initiative, the study exemplifies how methodological rigour and stakeholder engagement can facilitate meaningful adaptation of global frameworks. Given the international urgency around missed care and care fragmentation, these validated tools contribute not only to Italian nursing reform but also offer a transferable model for other countries seeking to embed relational, values‐based care into everyday practice.

The findings are particularly relevant given ongoing global concerns around missed nursing care and declining relational care standards (Kalisch 2006; Danielis et al. 2022). By providing a shared language and structured approach to fundamental care, the validated FoC tools have the potential to help nurses overcome systemic barriers, including task fragmentation and time pressure. The framework's emphasis on relational care is especially crucial in fostering meaningful interactions with patients and families—an essential but often overlooked component of high‐quality care (Mudd et al. 2020).

The analysis of expert interviews provided valuable insights into the perceived strengths of the framework. Experts noted that the FoC Framework encourages nurses to reflect critically on their practice, fostering a deeper understanding of their role in addressing patients' physical, psychosocial and relational needs. This aligns with existing literature emphasising the role of theory‐guided nursing practice in improving care quality (Younas and Quennell 2019).

The validated FoC tools carry important implications for education, clinical practice and research. In educational contexts, these tools can anchor theoretical instruction into real‐world nursing values, reinforcing reflection, critical thinking and patient‐centred practice. In clinical settings, they can support nurse‐led care planning, interdisciplinary communication and quality improvement initiatives. In research, the culturally adapted tools enable the systematic study of fundamental care practices across Italian healthcare environments and support cross‐national comparisons within the ILC network.

While the Framework and Practice Process offer substantial promise, several limitations must be acknowledged. Their effective implementation depends on contextual adaptation, institutional commitment and the availability of adequate training resources. Potential barriers include language differences, organisational inertia and competing clinical demands.

Future research should not only evaluate implementation outcomes and address local barriers but also explore how these tools might catalyse cultural change in the way fundamentals of care are taught, delivered and valued across diverse healthcare settings. Taken together, these findings demonstrate that cross‐cultural validation is not merely a technical process of translation, but a reflective negotiation of values, language and practice. The Italian adaptation reveals both the universal relevance of fundamentals of care and the essential role of localised meaning‐making in ensuring meaningful, context‐sensitive implementation.

## 7 Conclusion

This study highlights the importance of culturally adapting theoretical frameworks to ensure their relevance, clarity and usability across diverse healthcare systems. The validated Italian versions of both the Fundamentals of Care Framework and the FoC Practice Process offer a significant contribution to nursing practice and education in Italy. Together, they provide a structured, evidence‐informed foundation for delivering holistic, person‐centred care that integrates physical, psychosocial and relational dimensions.

Through expert review and user testing, Italian adaptations demonstrated strong conceptual equivalence, linguistic clarity and contextual fit. The FoC Framework serves as a theoretical guide for addressing core nursing responsibilities, overcoming barriers to fundamental care and promoting reflective, relational practice. The accompanying Practice Process offers a practical, step‐by‐step model that supports implementation in educational and clinical settings where structured guidance is critical.

Integrating these tools into Italian nursing curricula and clinical practice holds promise for improving care quality, patient safety and professional development. Future research should examine their impact on clinical outcomes, student learning and organisational culture, and assess their adaptability across different regions and international settings—further positioning the FoC Framework and Practice Process as global standards for fundamentals of nursing care.

## Author Contributions

**Annamaria Bagnasco:** conceptualization, methodology, resources, supervision. **Maura Lusignani:** methodology, resources, supervision. **Nicola Pagnucci:** conceptualization, methodology, writing – original draft. **Talita Sallai:** methodology, investigation, data curation, formal analysis, writing – original draft. **Gianluca Catania:** conceptualization, methodology, resources, supervision, writing – review and editing. **Francesca Napolitano:** investigation, data curation, formal analysis, writing – review and editing. **Alberto Dal Molin:** methodology, resources, supervision. **Beatrice Mazzoleni:** methodology, resources, supervision. **Simone Cosmai:** investigation. **Daniela Cattani:** investigation. **Laura Mansi:** investigation. **Doriana Montani:** investigation. **Andreina Zavaglio:** investigation. **Paola Sanvito:** investigation. **Chiara Cartabia:** investigation. **Letteria Consolo:** investigation. **Luca Giuseppe Re:** investigation. **Milko Zanini:** conceptualization, methodology, resources, supervision. **Loredana Sasso:** conceptualization, methodology, resources, supervision.

## Ethics Statement

This study was approved by the Territorial Ethics Committee of the Liguria Region (591/2021) as part of the study ‘Integrating the Fundamentals of Care Framework into Italian Nursing Education (FOC‐FORM Study)’. Confidentiality and anonymity were ensured by assigning codes to interview transcripts and anonymising questionnaire responses. No monetary compensation or incentives were provided for participation. Data was securely stored and used exclusively for research purposes, in compliance with data protection regulations. Participants were given opportunities to ask questions and seek clarification before data collection began. The study adhered to the principles outlined in the Declaration of Helsinki and relevant national ethical guidelines, ensuring the highest standards of research integrity. Permission to use and reproduce material from external sources was obtained, with relevant citations provided in the manuscript.

## Consent

All participants were fully informed about the study's purpose, procedures, potential risks and benefits before their involvement. Written informed consent was obtained from each participant to confirm their voluntary agreement to participate. For interviews requiring audio or video recording, verbal consent was additionally obtained at the beginning of the session. Participation in the study was entirely voluntary, with participants informed of their right to withdraw at any time without consequences.

## Conflicts of Interest

The authors declare no conflicts of interest.

## Supporting information


**Table S1:** jan70099‐sup‐0001‐TableS1.docx.

## Data Availability

The data that support the findings of this study are available from the corresponding author upon reasonable request.
