# Supplementary material for: Italian Version of the Fundamentals of Care Framework and the Fundamentals of Care Practice Process: A Comprehensive Validation Study
Source: J Adv Nurs. 2025 Jul 28;82(4):3449–63. doi: 10.1111/jan.70099 (PMC12994631; doi:10.1111/jan.70099)
Supplement: Supplementary file 1 — Table S1: jan70099‐sup‐0001‐TableS1.docx. [file JAN-82-3449-s001.docx]

# CONSOLIDATED CRITERIA FOR REPORTING QUALITATIVE RESEARCH (COREQ): A 32-ITEM CHECKLIST FOR INTERVIEWS AND FOCUS GROUPS

Table S1. Consolidated criteria for reporting qualitative studies (COREQ): 32-item checklist

| No | Item | Guide questions/description | Pag |
| --- | --- | --- | --- |
| Domain 1: Research team and reflexivity | | | |
| Personal Characteristics | | | |
| 1. | Interviewer/ facilitator | Which author/s conducted the interview or focus group? | 6 |
| 2. | Credentials | What were the researcher's credentials? E.g. PhD, MD | 6 |
| 3. | Occupation | What was their occupation at the time of the study? | 6 |
| 4. | Gender | Was the researcher male or female? | 6 |
| 5. | Experience and training | What experience or training did the researcher have? | 6 |
| Relationship with participants | | | |
| 6. | Relationship established | Was a relationship established prior to study commencement? | 6 |
| 7. | Participant knowledge of the interviewer | What did the participants know about the researcher? e.g. personal goals, reasons for doing the research | 6 |
| 8. | Interviewer characteristics | What characteristics were reported about the interviewer/facilitator? e.g. Bias, assumptions, reasons and interests in the research topic | 9 |
| Domain 2: study design | | | |
| Theoretical framework | | | |
| 9. | Methodological orientation and Theory | What methodological orientation was stated to underpin the study? *e.g. grounded theory, discourse analysis, ethnography, phenomenology, content analysis* | 4 |
| Participant selection | | | |
| 10. | Sampling | How were participants selected? *e.g. purposive, convenience, consecutive, snowball* | 6 |
| 11. | Method of approach | How were participants approached? e*.g. face-to-face, telephone, mail, email* | 6 |
| 12. | Sample size | How many participants were in the study? | 6 |
| 13. | Non-participation | How many people refused to participate or dropped out? Reasons? | n/a |
| Setting | | | |
| 14. | Setting of data collection | Where was the data collected? e*.g. home, clinic, workplace* | 6 |
| 15. | Presence of non-participants | Was anyone else present besides the participants and researchers? | 6 |
| 16. | Description of sample | What are the important characteristics of the sample? *e.g. demographic data, date* | 9 |
| Data collection | | | |
| 17. | Interview guide | Were questions, prompts, guides provided by the authors? Was it pilot tested? | Table 2 |
| 18. | Repeat interviews | Were repeat interviews carried out? If yes, how many? | n/a |
| 19. | Audio/visual recording | Did the research use audio or visual recording to collect the data? | 6 |
| 20. | Field notes | Were field notes made during and/or after the interview or focus group? | no |
| 21. | Duration | What was the duration of the interviews or focus group? | 6 |
| 22. | Data saturation | Was data saturation discussed? | n/a |
| 23. | Transcripts returned | Were transcripts returned to participants for comment and/or correction? | no |
| Domain 3: analysis and findingsz | | | |
| Data analysis | | | |
| 24. | Number of data coders | How many data coders coded the data? | 7 |
| 25. | Description of the coding tree | Did authors provide a description of the coding tree? | Table 5 |
| 26. | Derivation of themes | Were themes identified in advance or derived from the data? | n/a |
| 27. | Software | What software, if applicable, was used to manage the data? | n/a |
| 28. | Participant checking | Did participants provide feedback on the findings? | n/a |
| Reporting | | | |
| 29. | Quotations presented | Were participant quotations presented to illustrate the themes / findings? Was each quotation identified? e*.g. participant number* | Table 5 and 6 |
| 30. | Data and findings consistent | Was there consistency between the data presented and the findings? | Table 5 and 6 |
| 31. | Clarity of major themes | Were major themes clearly presented in the findings? | Table 5 and 6 |
| 32. | Clarity of minor themes | Is there a description of diverse cases or discussion of minor themes? | n/a |
